# Fine Particulate Matter (PM2.5) Disrupts Intestinal Barrier Function by Inducing Oxidative Stress and PI3K/AKT-Mediated Inflammation in Caco-2 Cells

**DOI:** 10.3390/ijms26178271

**Published:** 2025-08-26

**Authors:** Ruiwei Liao, Qianwen Zhang, Yao Lu, Feifei Huang, Wenjuan Cao, Ming Li, Lin Zhou, Yan Li

**Affiliations:** 1 School of Basic Medical Sciences, Guangzhou University of Chinese Medicine, China No. 232, East Waihuan Road, Guangzhou Higher Education Mega Centre, Guangzhou 510006, China; liaoruiwei@stu.gzucm.edu.cn (R.L.);; 2 School of Basic Medical Sciences, Guangdong Pharmaceutical University, China No. 280, East Waihuan Road, Guangzhou Higher Education Mega Centre, Guangzhou 510006, China; 3 School of Life Science and Biopharmaceutics, Guangdong Pharmaceutical University, China No. 280, East Waihuan Road, Guangzhou Higher Education Mega Centre, Guangzhou 510006, China

**Keywords:** PM2.5, intestinal permeability, inflammation, PI3K/AKT pathway, oxidative stress

## Abstract

Fine particulate matter (PM2.5) is an environmental factor that triggers gastrointestinal diseases. However, the effects of PM2.5 on intestinal function are not fully understood. This study established an environmental exposure cell model to explore PM2.5-induced intestinal permeability alteration and its mechanisms. Intestinal barrier permeability was evaluated via trans-epithelial electrical resistance (TEER) measurement and FITC–dextran paracellular penetration analysis, followed by detection of intercellular junction protein β-catenin and its coding gene CTNNB1. Expression of inflammatory cytokines (TNF-α, IL-6) and phosphorylation of PI3K and AKT were assessed using quantitative real-time polymerase chain reaction and Western blot, respectively. Reactive oxygen species (ROS) and malondialdehyde were measured using commercial kits to observe cellular oxidative stress. The results showed that PM2.5 impaired the intestinal barrier, as indicated by reduced TEER, increased FITC–dextran penetration, down-regulated expression of β-catenin and CTNNB1. Additionally, compared with the control, inflammatory cytokines and oxidative stress markers were significantly elevated after PM2.5 exposure. The ratio of p-PI3K/PI3K and p-AKT/AKT was also up-regulated in PM2.5-exposed Caco-2 cells. Pretreatment with PI3K inhibitor LY294002 and ROS scavenger NAC modulated β-catenin expression, reduced inflammation/ROS, and alleviated the hyperpermeability of Caco-2 cells. Thus, our results reveal that PM2.5 induces PI3K/AKT-mediated inflammation and ROS generation in Caco-2 cells, leading to intestinal barrier impairment.

## 1. Introduction

In recent years, air pollution has emerged as one of the most critical threats to human health. There is growing concern about its potential health impacts. Air pollution is predominantly linked to respiratory diseases, such as chronic bronchitis and chronic obstructive pulmonary disease [1]. Children, the elderly, and individuals with pre-existing lung conditions are particularly vulnerable [2,3]. As a major constituent of air pollution, PM2.5 is its main toxic component, characterized by its small particle size, large specific surface area, and high adsorption capacity for harmful chemicals [4,5]. It is capable of causing diseases independently or in combination with other factors, such as O_3_, ultraviolet, and even a high-fat diet [6,7,8]. Except respiratory diseases, researchers have linked PM2.5 exposure to an increasing number of adverse health outcomes, including increased cardiovascular mortality, accelerated atherosclerosis in postmenopausal women, type 2 diabetes mellitus (T2DM), skin-related diseases, and even increased risk of venous thromboembolism [9,10,11,12,13].

Recently, epidemiological studies have shown that deteriorating air quality increases the incidence of gastrointestinal disorders, including inflammatory bowel disease (IBD) and irritable bowel syndrome (IBS) [14,15]. Other research also reported that particulate matter exposure may increase the risk of appendicitis and pediatric gastroenteritis and even the rate of colorectal cancer [16,17,18]. A cohort study reported that an increase of 1 μg/m^3^ in PM2.5 translates to an increase in the IBS incidence rate of about 0.02 cases per 100 person-years, which firmly proved that environmental pollutants are associated with IBS [19]. Jie Chen et al. found that ambient pollution exposure was associated with an increased risk of enterotomy and all-cause mortality among individuals with IBD, highlighting the important role of environmental health in improving the prognosis of IBD [20]. Chong Fu et al. underscored the potential involvement of PM2.5 in ulcerative colitis (UC) pathogenesis [21]. These findings highlight that PM2.5 may enter and cause harm to the gastrointestinal tract (GI). In 2004, Möller W et al. demonstrated that PM2.5 can be phagocytosed by alveolar macrophages, transported out of the lungs via ciliary beating in the respiratory mucus layer, and delivered to the oropharynx for subsequent swallowing into the GI [22]. Additionally, consumption of contaminated food or water facilitates direct uptake of PM2.5 into the intestinal tract [23]. A recent study proved that chronic PM2.5 exposure could induce intestinal disturbance via alternation of the gut microbiome [24]. However, most published papers related to PM2.5 have focused on cardiovascular and airway diseases, with very few studies examining the effects of PM2.5 in the gastrointestinal system.

The integrity of the intestinal epithelial barrier is critical for maintaining the physiological function of the gut. It is reported that toxicants and some enzymes can induce intestinal diseases by interfering with intestinal epithelial cells or their intercellular junctions, whereas protecting epithelial cells or restoring the junction will decrease the toxic effects [25]. The intestinal epithelial barrier is primarily dependent on junctional complexes within the protein network, including tight junctions (TJs), adherens junctions (AJs), and desmosomes [26]. As an armadillo-repeat protein in AJs, β-catenin binding with transmembrane cadherins and α-catenin is a vital step in adherens junction formation, crucial for intercellular adhesive function and maintaining intestinal epithelial barrier integrity [27,28]. These junction proteins are usually decreased in IBS. Wenxin Xia et al. demonstrated that oral hyperoside administration maintained the intestinal barrier integrity of dextran sulfate sodium (DSS)-induced colitis mice by elevating the expression of TJs and AJs, including E-cadherin and β-catenin, and the same results were obtained in lipopolysaccharide (LPS)-induced human colonic epithelial cells [29]. Several other studies also proved the key roles of β-catenin in maintaining the intestinal mucosal barrier function [30,31,32]. Arong Gaowa et al. reported that the Wnt/β-catenin and focal adhesion kinase signaling pathways were activated by irisin in intestinal epithelial cells, thereby promoting intestinal epithelial self-renewal under normal homeostatic conditions and intestinal epithelial regeneration upon damage [33]. Conversely, vitamin D deficiency in maternal mice was shown to inhibit the Wnt/β-catenin signaling pathway, down-regulate small intestinal tight junction protein expression, and ultimately increase intestinal permeability in offspring [34]. In the intestinal mucosa of patients with ulcerative colitis (UC) and Crohn’s disease (CD), decreased β-catenin expression and loss of TJ proteins are observed [35]. However, data on PM2.5 affecting intestinal barrier function and its regulation in the expression of β-catenin remain scarce.

Inflammation plays crucial roles in diseases characterized by barrier dysfunction. Researchers have reported that the blood–brain barrier (BBB) is highly sensitive to inflammatory stimuli, such as lipopolysaccharide (LPS) and TNF-α, which disrupts BBB function via multiple pathways [36,37]. Blamire et al. reported that intracerebral injection of interleukin-1β (IL-1β) induces inflammation, leading to increased BBB permeability [38]. Furthermore, Xian et al. demonstrated that PM2.5 impairs human nasal epithelial barrier function by reducing tight junction protein expression and increasing proinflammatory cytokine release [39]. In a chronic preclinical stress model in mice, researchers found that it had increased levels of cytokine IL-6 and a decrease in tight junction proteins in the colon, as demonstrated by the inverse correlation between IL-6 levels and occludin expression [40].

Oxidative stress is recognized as one of the core pathological mechanisms underlying the development of intestinal diseases, triggering intestinal mucosal injury, inflammatory cascade, and barrier dysfunction [41,42]. For example, acute and chronic GI disorders in humans and animal models are both characterized by increased reactive oxygen species (ROS) production or decreased counteracting antioxidants [43,44,45]. Studies have demonstrated that Ganjiang Huangqin Huanglian Renshen Decoction effectively alleviates the DSS-induced UC symptoms in mice by reducing oxidative stress [46]. Furthermore, an experiment demonstrated that Jiawei Gegen Qinlian Decoction ameliorates UC by mitigating inflammation, reducing oxidative stress, and enhancing intestinal barrier function in vitro and in vivo [47].

The phosphatidylinositol 3-kinase (PI3K)/protein kinase B (PKB/AKT) pathway is a major signaling cascade that significantly contributes to the regulation of various pathophysiological processes such as metabolism, oxidative stress, and immune inflammation [48,49,50,51]. The PI3K/AKT signaling pathway can activate the NF-κB to promote the expression of proinflammatory cytokines such as IL-6 and TNF-α [52]. A recent study reported that inter-alpha-trypsin-inhibitor heavy chain 4 induced proinflammatory cytokine production by regulating PI3K/Akt signaling, thus aggravating the progression of arthritis-like symptoms in the rat model [53]. Dan Xie et al. also proved suppressing PI3K/Akt activation may act against acute pancreatitis by reducing the production of inflammatory factors [54]. In addition, experiments demonstrated that inhibition of the PI3K/AKT signaling pathway blocked cell apoptosis and inflammation, which significantly ameliorated intestinal barrier dysfunction and the clinical symptoms of IBS [55]. Yang Liu et al. reported that platycodon grandiflorus polysaccharides combined with hesperidin exerted the synergistic effect of relieving ulcerative colitis in mice by modulating PI3K/AKT signaling pathways [56]. Qian Li proved citri reticulatae pericarpium protected intestinal mucosa by inhibiting inflammation through the PI3K/AKT pathway [57]. Conversely, glutamine deprivation over-activated the PI3K/AKT pathway, resulting in hyperpermeability of Caco-2 cells [58]. All these results indicate PI3K/AKT plays an important role in sustaining the physiological function of intestinal mucosa.

Due to the high costs and prolonged experimental period of a traditional animal study, in vitro cell models are usually used in the toxicological assessment of toxins in the intestinal barrier [59]. During the past few decades, different intestinal epithelial cell lines from various animal species as well as from human beings have been used to assess the effects of drugs and toxins on the permeability of the intestinal epithelium. Among them, the Caco-2 cell line is accepted as a reference model. The Caco-2 cell, derived from human colon adenocarcinoma cells, is capable of differentiating into an enterocyte-like phenotype, exhibiting morphological and functional characteristics analogous to normal small intestinal epithelial cells [60]. During culturing, they can express the tight junction (TJ) proteins between adjacent cells, so this Caco-2 cell model is a commonly used in vitro model to study the intestinal permeability in many studies [61]. Furthermore, this model is operationally straightforward, highly reproducible, and facilitates targeted investigations into intestinal barrier impairment, functioning as a standardized platform for drug screening, too [62]. Consequently, in the present study, we aim to establish a Caco-2 cell model to explore the toxic effects and underlying mechanisms of PM2.5-induced intestinal hyperpermeability.

In the present study, a PM2.5 exposure intestinal epithelial cell model was used to discuss the toxic effects and cellular mechanisms by which PM2.5 impacts intestinal barrier function. The intestinal epithelial permeability and junctional protein β-catenin expression level were detected to assess the intestinal barrier function. Then, the production of inflammatory cytokines and ROS and the activation of the PI3K/AKT pathway were measured to reveal the underlying mechanisms. Furthermore, the PI3K inhibitor LY294002 and ROS scavenger NAC were also used for mechanism research.

## 2. Results

### 2.1. Cell Viability Assay

Caco-2 cells were treated with 6.25, 12.5, and 25 μg/mL of PM2.5 for 24 h, and cell viability was evaluated via the CCK-8 assay. The results showed that at a concentration under 25 μg/mL, PM2.5 did not exhibit significant cytotoxicity on Caco-2 cells.

### 2.2. PM2.5 Increases Permeability of Caco-2 Cells

**Figure 1:**
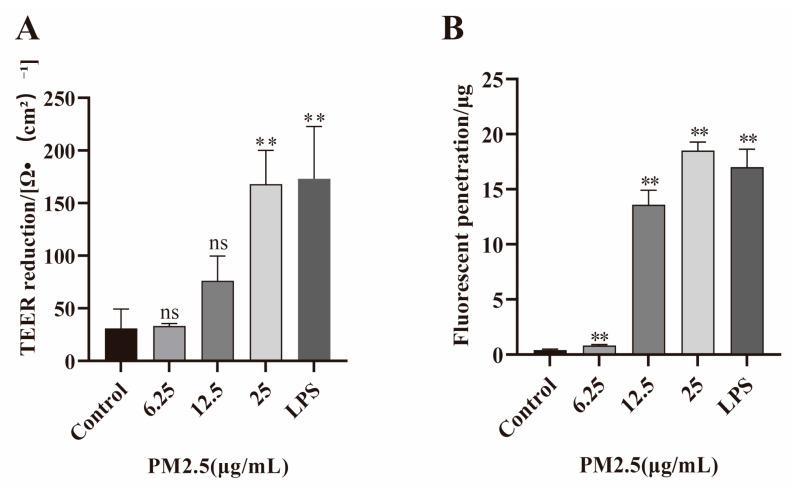
Effects of PM2.5 on Caco-2 cell permeability. Caco-2 cells were treated with 6.25, 12.5, 25 μg/mL PM2.5 and LPS (100 ng/mL, as positive control). (**A**) Reduction in TEER. (**B**) Increase in FITC–dextran penetration was seen in PM2.5 treatment group. Data presented as means ± SD, *n* = 3. Ns, non-significant. ** *p* < 0.01 was used for comparison with untreated cells in the control group.

Increased cell permeability is associated with GI-related diseases. TEER and FITC–dextran penetration assays were performed in this study to detect the cell permeability [63]. A decrease in intercellular resistance indicates an increase in cell permeability. Caco-2 cells were exposed to non-cytotoxic doses of PM2.5 (6.25, 12.5, and 25 μg/mL), and the TEER was measured using a transmembrane resistance meter. Cells treated with phosphate-buffered saline (PBS) were used as the control. The results (as shown in Figure 1A) showed an obvious decrease in TEER in PM2.5-treated Caco-2 cells. To further confirm these results, FITC–dextran penetration assay was performed, and a higher fluorescent penetration was observed in the PM2.5-exposed group (as shown in Figure 1B). Together, our results indicate that PM2.5 can damage intestinal barrier function and enhance its permeability. Given that 25 μg/mL PM2.5 was non-cytotoxic and produced significant barrier dysfunction (*p* < 0.01, 25 μg/mL group vs. 6.25 and 12.5 μg/mL group, respectively), this concentration was selected for subsequent mechanistic studies.

### 2.3. PM2.5 Reduces β-catenin and Its Encoding Gene CTNNB1 Expression

**Figure 2:**
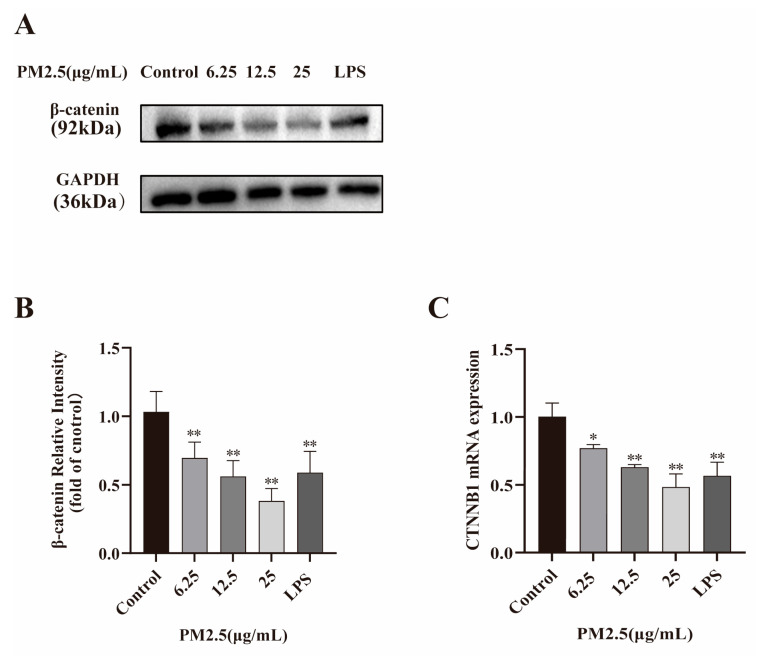
PM2.5 down-regulated β-catenin expression in Caco-2 cells. LPS (100 ng/mL) was used as a positive control. (**A**,**B**) β-catenin expression was assessed by Western blot. (**C**) CTNNB1 expression was evaluated by qRT-PCR. Data presented as means ± SD, *n* = 3. * *p* < 0.05, ** *p* < 0.01 was used for comparison with untreated cells in the control group. Ns means not significant.

To explore the potential mechanisms of PM2.5-induced barrier dysfunction, the expression of the junctional protein β-catenin and its coding gene CTNNB1 was analyzed via Western blot and qRT-PCR, respectively. The results proved the mRNA and protein expression levels of β-catenin in Caco-2 cells were both markedly down-regulated by PM2.5 treatment (Figure 2). Taken together, the above results indicate that PM2.5 diminished the key junction protein β-catenin expression, finally resulting in intestinal barrier integrity disruption.

### 2.4. PM2.5 Induced Inflammatory Factor Expression

**Figure 3:**
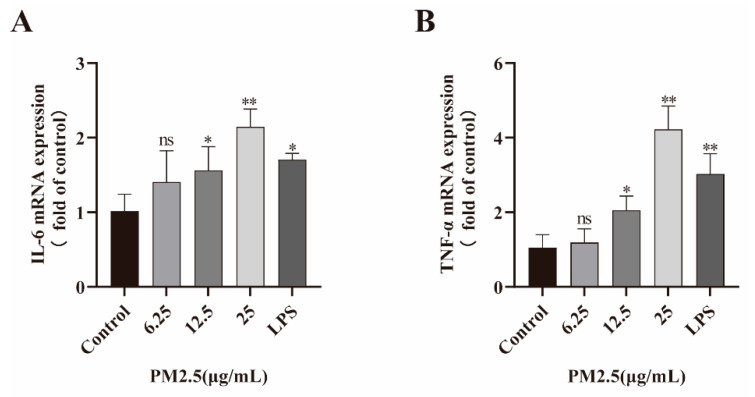
PM2.5 increases the production of IL-6 and TNF-α in Caco-2 cells. Cells were treated with PM2.5 or LPS, respectively, and inflammatory cytokine expression was assessed by qRT-PCR. LPS (100 ng/mL) was used as a positive control. (**A**) The expression of IL-6. (**B**) The expression of TNF-α. Data presented as means ± SD, *n* = 3. Ns, non-significant. * *p* < 0.05, ** *p* < 0.01 were used for comparison with untreated cells in the control group.

Local inflammatory response is an important cause of intestinal barrier function damage. Therefore, Caco-2 cells were incubated with PM2.5 (6.25, 12.5, and 25 μg/mL) and LPS, and the inflammatory cytokine (IL-6, TNF-α) expression was measured with qRT-PCR. As shown in Figure 3, PM2.5 treatment induced the generation of IL-6, TNF-α in Caco-2 cells, especially at a PM2.5 concentration of 25 μg/mL.

### 2.5. PM2.5 Activates PI3K/AKT Signaling Pathway

**Figure 4:**
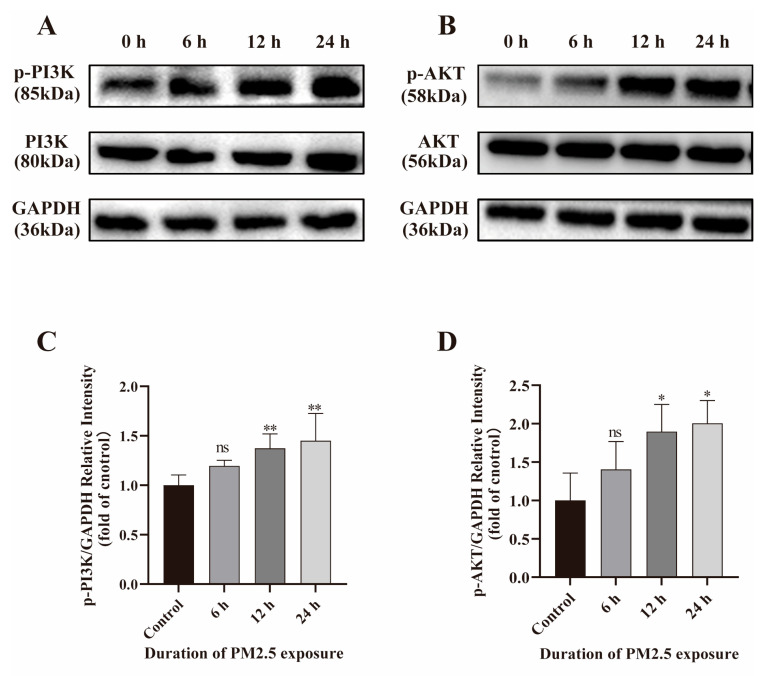
Effects of PM2.5 on the activation of PI3K/AKT pathway. (**A**–**D**) The phosphorylation of PI3K and AKT in PM2.5-exposed Caco-2 cells was assessed by Western blot. Data presented as means ± SD, *n* = 3. Ns, non-significant. * *p* < 0.05 and ** *p* < 0.01 were used for comparison with untreated cells in the control group.

Western blot was performed to assess the activation of the PI3K/AKT pathway. As presented in Figure 4A,B, the results showed the phosphorylation levels of PI3K and AKT were markedly up-regulated with PM2.5 treatment. Intensity analysis (Figure 4C,D) indicated the ratios of p-PI3K/PI3K and p-AKT/AKT were also increased. These results demonstrate that PM2.5 may induce inflammatory responses by enhancing phosphorylation of the PI3K/AKT.

### 2.6. LY294002 Against PM2.5-Induced Intestinal Hyperpermeability

**Figure 5:**
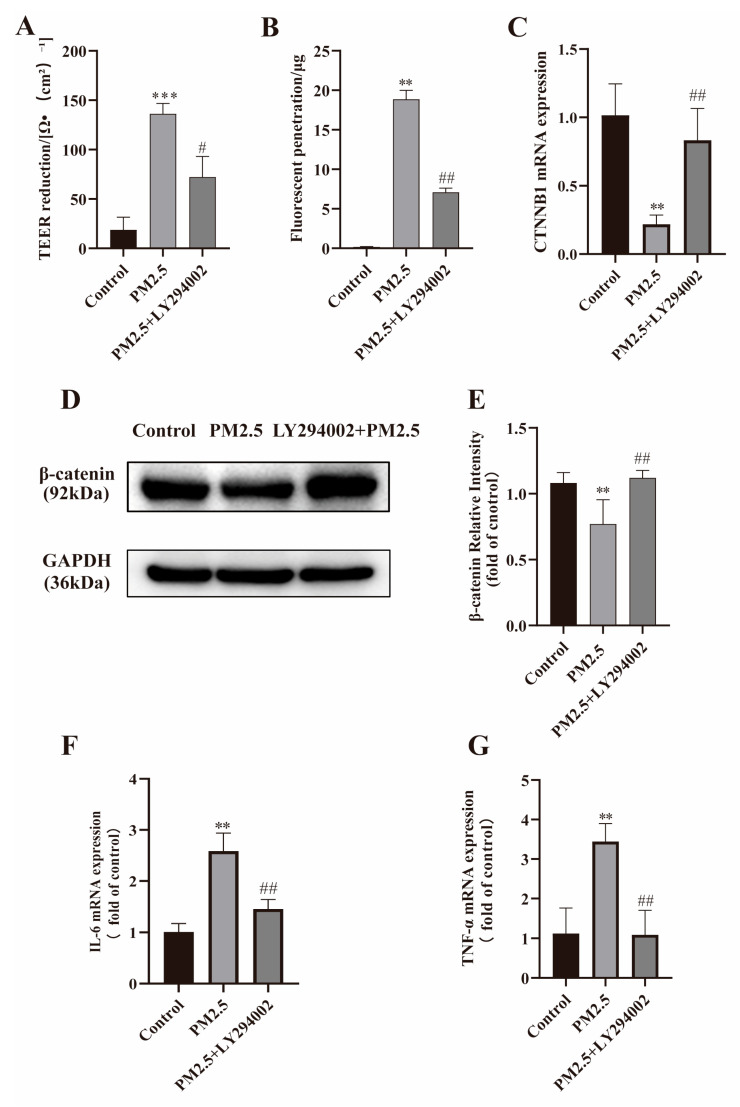
LY294002 against PM2.5-induced intestinal hyperpermeability by up-regulating β-catenin expression and inhibiting inflammation response. Caco-2 cells were pretreated with LY294002 (10 μmol/L) for 2 h, followed by treatment with PM2.5 (25 μg/mL). (**A**) Reduction in TEER. (**B**) FITC–dextran penetration. (**C**–**E**) Expression of β-catenin and its encoding gene CTNNB1 was detected as described in Methods. (**F**,**G**) Expression of TNF-α and IL-6 was detected by qRT-PCR. Data presented as means ± SD, *n* = 3. ** *p* < 0.01 and *** *p* < 0.001 were used for comparison with control group. ^#^ *p* < 0.05 and ^##^ *p* < 0.01 were used for comparison with PM2.5 group.

The above results indicate PM2.5 may decrease β-catenin expression and induce gut hyperpermeability by inducing inflammation through the PI3K/AKT pathway. LY294002, a PI3K inhibitor, was used to prove this hypothesis. Caco-2 cells were pretreated with LY294002 (10 μM) for 2 h and then exposed to PM2.5 (25 μg/mL). The results showed Caco-2 cell permeability was restored, as indicated by increased TEER and decreased FITC–dextran penetration (Figure 5A,B). Consistent with this, it was also observed that the expression of junction protein β-catenin and its coding gene CTNNB1 was up-regulated in the LY294002 pretreatment group (Figure 5C–E). Additionally, compared with PM2.5 exposure alone, TNF-α and IL-6 expression levels in the LY294002 pretreatment group were obviously decreased (Figure 5F,G). Altogether, these results clearly proved that inflammation mediated by the PI3K/AKT pathway is involved in the PM2.5-induced hyperpermeability in Caco-2 cells.

### 2.7. PM2.5-Induced Oxidative Stress in Caco-2 Cells

**Figure 6:**
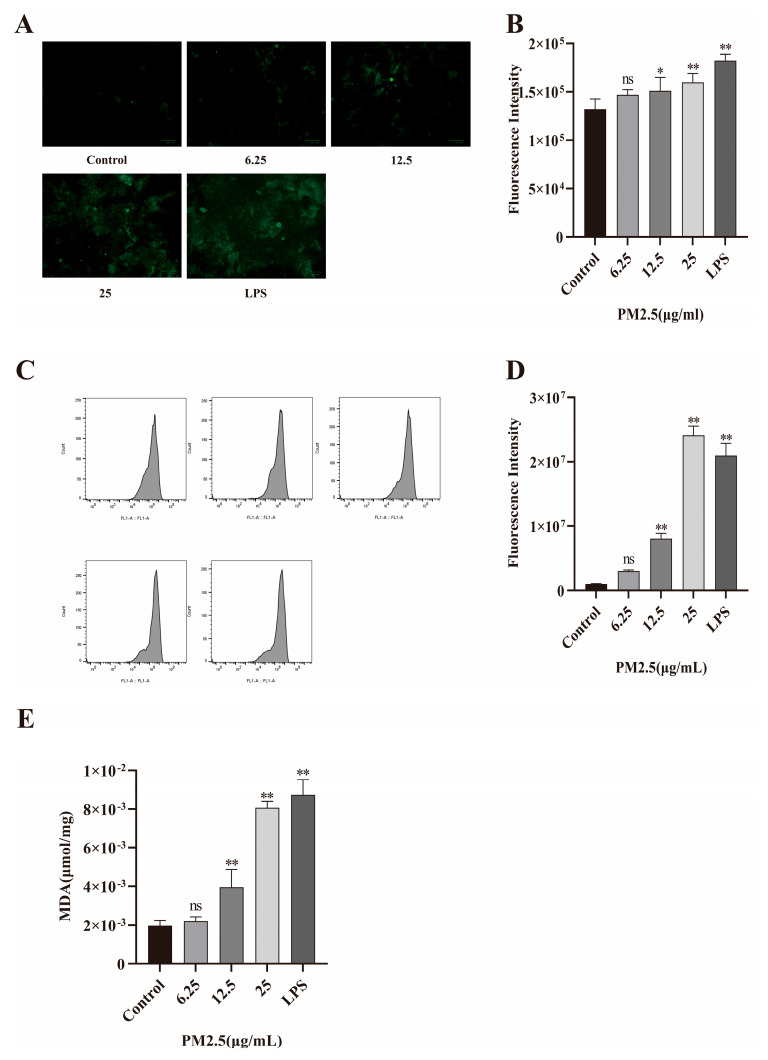
PM2.5 elevates oxidative stress in Caco-2 cells. LPS (100 ng/mL) was used as a positive control. (**A**,**B**) Visualization of ROS using fluorescence microscopy and analyses by Image J. (**C**,**D**) Detection of intracellular ROS levels in Caco-2 cells by flow cytometry. (**E**) Malondialdehyde production in cells after PM2.5 treatment. Data presented as means ± SD, *n* = 3. Ns, non-significant. * *p* < 0.05 and ** *p* < 0.01 were used for comparison with control group.

Oxidative stress also plays a vital role in intestinal barrier damage. Therefore, Caco-2 cells were treated with PM2.5 or LPS (as positive control), respectively. Then, the levels of ROS and MDA were measured. The results from DCFH-DA fluorescence staining and flow cytometry both proved excessive ROS production in the PM2.5-exposed Caco-2 cells, as shown in Figure 6A–D. MDA is a product formed by free radicals with cell membranes and is a commonly used indicator for evaluating oxidative stress. As seen in Figure 6E, MDA also increased after PM2.5 exposure.

### 2.8. NAC Against PM2.5-Induced Barrier Dysfunction

**Figure 7:**
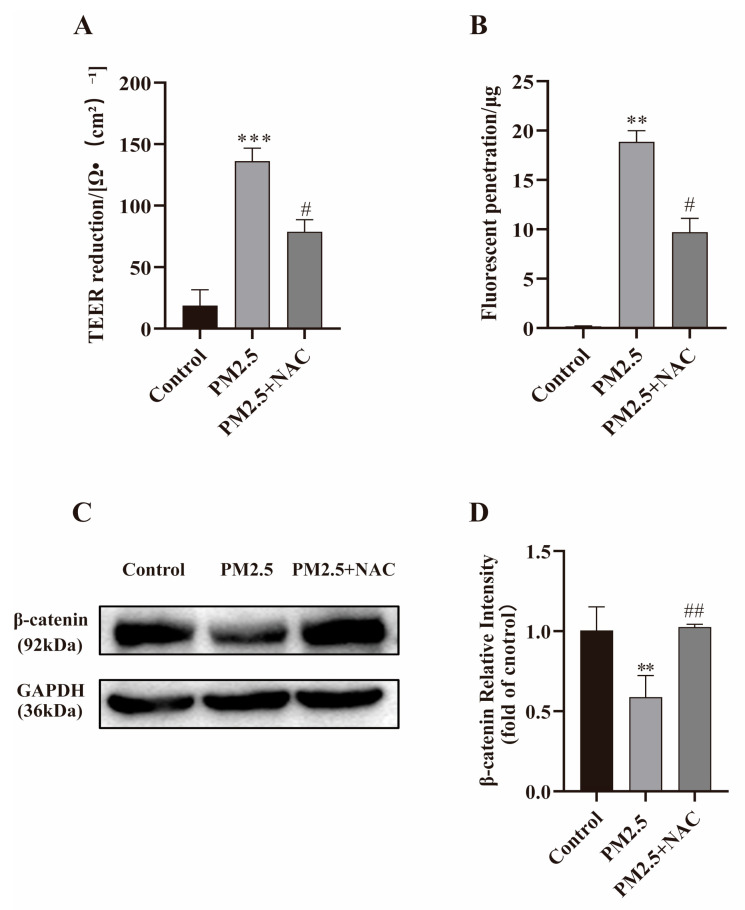
NAC inhibits PM2.5-induced increases in intestinal permeability and up-regulates β-catenin expression. (**A**) Reduction in TEER. (**B**) FITC–dextran permeability. (**C**,**D**) β-catenin expression was measured in Caco-2 cells treated with NAC and PM2.5 (25 μg/mL). Data presented as means ± SD, *n* = 3. ** *p* < 0.01 and *** *p* < 0.001 were used for comparison control group. ^#^ *p* < 0.05 and ^##^ *p* < 0.01 were used for comparison with PM2.5 group.

NAC, a ROS scavenger, was used to confirm the relationship between oxidative stress and barrier dysfunction induced by PM2.5. Caco-2 cells were treated with PM2.5 (25 μg/mL) alone or combined with NAC (50 mM). Compared with the PM2.5 exposure group, NAC effectively increased TEER and decreased the fluorescence penetration along with the up-regulated expression of β-catenin (shown in Figure 7). These results strongly suggested induction of ROS is also one of the key factors contributing to PM2.5-induced intestinal barrier dysfunction.

## 3. Discussion

Recently, a few studies have linked air pollution exposure to the onset and progress of gastrointestinal diseases, but the mechanisms are still unknown. In the present study, we found that PM2.5 exposure impaired intestinal barrier function, as indicated by decreased TEER, increased penetration of FITC–dextran, and disruption of junction protein β-catenin expression. Exposure to PM2.5 results in activation of the PI3K/AKT pathway, the overproduction of inflammatory cytokines and ROS in intestinal epithelial cells, which are responsible for barrier breakdown. Notably, pretreatment with PI3K inhibitor LY294002 and ROS scavenger NAC effectively reduced the production of inflammatory cytokines and oxidative stress products, thereby preventing PM2.5-induced β-catenin disruption and protecting intestinal barrier function. Then, our results firmly proved PI3K/AKT-mediated inflammation response and excessive ROS production are involved in PM2.5-induced intestinal barrier dysfunction under non-cell-death-inducing concentrations. To the best of our knowledge, this is the first study to unveil the underlying mechanisms, and it provides new insight into the adverse health effects of PM2.5 on intestinal mucosa.

PM2.5 exposure may increase the risk of intestinal diseases in humans. Data from the UK Biobank’s large-scale prospective cohort study reveal that long-term exposure to PM10 and PM2.5 was associated with IBS, and PM2.5 exerted a greater adverse effect than PM10 [64]. Additionally, a meta-analysis including 10 studies from North America and Asia found a significant association between PM2.5 exposure and the risk of colorectal cancer [65]. Recently, Zihan Ran et al. reported that chronic PM2.5 exposure may induce intestinal disturbance via inflammation and dysregulation of the gut microbiome [66]. Although increasing studies have suggested that PM2.5 exposure may induce intestinal diseases, the pathogenic mechanisms remain unclear. Our study demonstrated that PM2.5 exposure leads to a significant increase in intestinal permeability, which may be one of the potential mechanisms underlying PM2.5-induced intestinal diseases. Furthermore, it has also been proved by researchers that PM2.5 can increase permeability in pulmonary microvascular endothelial cells, corneal epithelial cells and BBB, through IL-6/HIF-1α, PI3K/AKT and other pathways. These results suggest that PM2.5 exposure not only enhances intestinal permeability but also exerts a similar effect in other cells.

Disruption of the mucosa layer and abnormal expression of junction proteins may lead to intestinal permeability damage, thereby accelerating the progression of various gastrointestinal diseases [67]. In permeability-related studies, junction proteins such as ZO-1 and β-catenin are commonly used as indicators. For instance, in studies on nasal mucosa, Zhao et al. demonstrated that PM2.5 down-regulates the expression of tight junction proteins such as ZO-1, thereby disrupting the nasal epithelial barrier [68]. Among adherens junction-associated proteins, β-catenin has received particular attention. β-catenin binding to cadherins is a key step in adherens junction formation, through which adjacent cells closely adhere to each other, ultimately sealing off the paracellular space and forming the intestinal barrier [69]. Yalan Dong et al. demonstrated that Berberine protects DSS-induced colitis primarily by maintaining the structural and functional integrity of the intestinal barrier via the Wnt/β-catenin pathway [70]. Our observation proves treatment of Caco-2 cells with PM2.5 leads to decreased β-catenin and its coding gene CTNNB1 expression, which results in a decrease in the integrity of the intestinal barrier. These findings confirm the critical role of β-catenin in PM2.5-related intestinal disease pathogenesis and suggest that protecting β-catenin may emerge as an effective therapeutic strategy. Going forward, we will conduct detection of other junction proteins to enhance the persuasiveness of the results.

It has been confirmed that in inflammation, serving as the body’s defensive responses to injury or toxins, its dysregulation or chronicity is the common pathological basis of various diseases. Studies have demonstrated that long-term exposure to PM2.5 induces endothelial and systemic inflammation, potentially leading to multi-system organ damage [71]. PM2.5 tends to induce intestinal diseases by triggering inflammatory responses, too. Our previous study demonstrated that short-time exposure to PM2.5 induced hepatic inflammation and finally resulted in insulin resistance [72]. An animal study showed that PM2.5 exposure could lead to inflammation in the trachea and lung [73]. Additionally, Yan Wang et al. reported that PM2.5 induced enterocyte apoptosis by activating the NF-κB and MAPK pathways, thereby promoting the release of inflammatory cytokines [74]. Our study found that PM2.5 exposure induced a significant increase in the production of inflammatory cytokines (IL-6, TNF-α) in Caco-2 cells, which is consistent with the aforementioned findings.

The PI3K/Akt signaling pathway has been shown to participate in the activation of inflammation [75,76]. Several studies proved that intestinal epithelial permeability is affected by PI3K/AKT pathway activation. In UC, AKT activation promotes inflammatory responses in the colon and facilitates the survival of inflammatory cells by its activated form [77]. Tiantian Wang et al. proved miR-124 may decrease inflammatory cytokine production and promote M2 macrophage polarization by suppressing the PI3K/Akt pathway [78]. Our experiments not only showed PM2.5 induced inflammatory cytokine production and activated the PI3K/Akt signaling pathway but also demonstrated that PI3K inhibitor LY294002 pretreatment effectively reduced TNF-α and IL-6 expression, attenuated the β-catenin disruption and alleviated the intestinal epithelial barrier damage, which indicated that PM2.5 exposure caused intestinal hyperpermeability may be dependent on the PI3K/AKT-mediated inflammatory response. Our results are also consistent with previous studies. These studies found LY294002 up-regulates junction protein Cx43 expression and mitigates injury of intestinal epithelial cells by irradiation [79].

Oxidative stress is widely recognized as a central mechanism of PM2.5-mediated toxic effects [80]. Both in vitro and in vivo experimental studies have reported increased ROS production in response to PM2.5 exposure, which caused varying degrees of oxidative injury to the lung and liver of mice [81]. Zhou Du et al. demonstrated that PM2.5 induces hepatic injury and fibrosis by triggering excessive ROS production in HepG2 cells [82]. In the present study, we confirmed PM2.5 exposure caused a marked increase in ROS and MDA in Caco-2 cells. Moreover, our study also found that ROS scavenger NAC exerted a protective effect against PM2.5-induced intestinal hyperpermeability, which advanced our understanding of the role that oxidative stress plays in intestinal diseases. Reports from Zhicong Hong et al. and several other researchers also indicated PM2.5 induced epithelial barrier dysfunction in the nose, lung and brain through ROS formation [83,84,85,86]. These findings highlight PM2.5 causes toxic effects via ROS, suggesting that a possible strategy to protect the epithelial barrier is to block the generation of ROS with antioxidant drugs.

Notably, inflammation and oxidative stress are not independent physiological processes but are closely interrelated. For instance, genital tract inflammation exacerbates oxidative stress, which in turn recruits and activates immune cells at inflamed sites [87,88]. IBD, a gastrointestinal inflammation-related disease, is also associated with increased local ROS production [89,90]. Similarly, PM2.5-induced lung injury is characterized by marked release of inflammatory cytokines and excessive accumulation of ROS [91]. In this study, we proved that PM2.5 induced oxidative stress and inflammation synergistically in Caco-2 cells, leading to intestinal barrier dysfunction.

This study is not without limitations. First, standard PM2.5 reference material was used in our research, and its chemical composition and physical properties may differ from real-world-derived PM2.5, so the toxic effects of PM2.5 collected from different areas need to be discussed. Meanwhile, PM2.5 is a complex mixture of multiple substances rather than a single entity. Our study does not identify which specific PM2.5 components contribute to intestinal barrier integrity impairment. Thus, determining the effects of individual PM2.5 components on PM2.5-induced gut barrier dysfunction represents a new challenge. Second, the key to an organism maintaining normal physiological functions lies in the integrated network formed by interconnected pathways and systems, rather than the simple superposition of an isolated and single pathway or mechanism. Thus, there may exist potential crosstalk between the PI3K/AKT pathway and oxidative stress. However, in our study, these pathways were investigated independently, and their crosstalk and regulatory mechanisms warrant further investigation in the future. At the same time, the upstream and downstream signals of PI3K/AKT also need to be discussed. TLRs and EGFR are commonly considered triggers for PI3K/AKT activation. While NF-κB and MAPKs work as targets following PI3K/AKT, the role of these molecules in PM2.5-induced intestinal dysfunction will be another key concern in our future study [92,93]. Furthermore, only an in vitro Caco-2 cell model was used in the present study to investigate the toxic effects of PM2.5. Therefore, in vivo studies (e.g., PM2.5-inhaled mice model) are warranted to further elucidate the effects and specific mechanisms by which PM2.5 disrupts gut epithelial barriers. Finally, although PM2.5 concentrations and overall air pollution have declined significantly since the 21st century, the incidence of non-cancerous gastrointestinal diseases has still surged up to now. This phenomenon indicates that there exist cofactors capable of enhancing the pathogenicity of PM2.5, among which obesity and diabetes mellitus stand as prominent examples. Thus, it is necessary to prioritize multi-factorial co-pathogenesis in the following research.

In this study, we highlight the critical roles of oxidative stress, inflammatory cytokines, and the PI3K/AKT signaling pathway in PM2.5-induced intestinal epithelial barrier damage. Our study also demonstrated that LY294002 and NAC protected β-catenin, thereby ameliorating PM2.5-induced intestinal hyperpermeability, which suggested that drugs targeting these related molecules represent potential effective strategies for preventing and treating PM2.5-associated intestinal barrier injury. Currently, gut microbiota has emerged as an influential factor affecting human health and disease. It has been proved by many studies that modulation of the gut microbiota can protect intestinal epithelial cells and regulate the development of related diseases [94]. This finding provides a new perspective for our subsequent research on protective approaches against PM2.5-induced intestinal barrier damage. This might become the main focus due to the limited efficacy of current preventive and therapeutic approaches for preventing the toxic effects of PM2.5.

## 4. Materials and Methods

### 4.1. Preparation of PM2.5

PM2.5 (Cat. No. SRM1649b) was purchased from the National Institute of Standards and Technology (NIST, Gaithersburg, MD, USA). It is collected from atmospheric particulate matter in Washington, DC, USA, and is widely used as standard reference material in PM2.5-related toxicological research [95]. Using DLS and SEM, our previous research revealed that the particle size of SRM-1649b was in fact below 2.5 μm (ranging from 349.5 nm to 1.755 µm) and enabled its use as a PM2.5 reference [72]. PM2.5 was suspended in PBS to prepare a 25 mg/mL suspension and stored at −20 °C for future use. 

### 4.2. Materials

A rabbit polyclonal antibody against β-catenin was purchased from ThreeEagles (Wuhan, China). GAPDH monoclonal antibody was obtained from ABclonal (Boston, MA, USA). The PI3K and p-PI3K antibodies were purchased from Affinity Biosciences (Changzhou, China). Akt antibody and p-Akt antibodies were purchased from Abcam (Cambridge, UK). Fluorescein Isothiocyanate-labeled Dextran (FITC-dextran, 4 kDa) was purchased from Merck (Kenilworth, NJ, USA). ROS assay kit, MDA assay kit and 2′,7′-dichlorodihydrofluorescein diacetate (DCFH-DA) were obtained from Beyotime Biotechnology (Shanghai, China). LY294002 and NAC were obtained from Sigma-Aldrich (St. Louis, MO, USA) and diluted with PBS to prepare a 1000X stock solution and stored at −20 °C, and appropriate volume was added to cell culture to obtain working concentration when used in the experiment. The working concentration of LY294002 and NAC is 10 μM and 50 mM, respectively.

### 4.3. Cell Culture and Viability Assay

Human colorectal adenocarcinoma cells (Caco-2) were obtained from Meilun Biotechnology Company (Dalian, China) and cultured in a humidified incubator at 37 °C with 5% CO_2_. The culture medium used was DMEM (Meilun, Dalian, China), supplemented with 20% fetal bovine serum (Shenghang, Nanjing, China) and 1% penicillin-streptomycin (Solarbio, Beijing, China). The culture medium was replaced 3-4 times per week, and cells in logarithmic growth phase were used in the following studies.

Cell viability was assessed using the commercial CCK-8 kit (Meilun, Dalian, China). Briefly, cells were seeded in 96-well plates at a density of 1 × 10^4^ cells per well for 24 h. Then, the incubated cells were treated with different concentrations of PM2.5 for 24 h, while equal volume of PBS was added to cells as control. Subsequently, cell viability was measured in accordance with the instructions of the CCK-8 kit, and absorbance at 450 nm was assessed using a microplate reader (Molecular Devices, San Jose, CA, USA). Cell viability was denoted as a percentage of the control value.

### 4.4. Measuring TEER and the Penetration of FITC–Dextran

The Caco-2 cells at a density of 5 × 10^4^ cells per well were seeded into a Transwell insert (pore size 0.4 μm, Corning; Costar, Cambridge, MA, USA). The TEER of the cells was measured using a transmembrane resistance meter (Merck, Kenilworth, NJ, USA), as described in a previous study [96]. Briefly, 600 μL PBS was placed at the bottom, and then measurements were taken on day 1, day 3 and every day up to day 14. The experimental data were presented in terms of resistance values. An insert without cells was used as a blank, and the measurement of the blank was subtracted from each sample reading in every experiment. The TEER measurements were also performed when the cells were exposed to PM2.5, NAC, LY294002, PM2.5 + LY294002 or PM2.5 + NAC for 24 h. TEER was denoted as Ω·cm^2^ = (R_measurement_ − R_blank_) × A, where R represents the resistance value and A denotes the area of the Transwell membrane.

FITC–dextran penetration assay was performed to measure the paracellular permeability of the Caco-2 cells. Cells were cultured in 24-well Transwell inserts. After being treated with different concentrations of PM2.5 with or without NAC/LY294002 for 24 h, FITC–dextran was added to the cells in apical chamber and incubated at 37 ˚C for 2 h. Then, the apical and basolateral fluids were collected, and the amount of FITC–dextran that transgressed the cell layer was measured using a microplate reader. The apparent permeability coefficient (Papp) refers to the macroscopic permeability of substances across monolayer cells under specific experimental conditions. Papp was denoted as cms^−1^ = ΔQ/(ΔtACo), where ΔQ is the fluorescence transmittance within Δt, A (cm^2^) is the membrane area, and Co is the initial concentration of fluorescence on the top surface of Caco-2 cells.

### 4.5. Quantitative Real-Time Polymerase Chain Reaction (qRT-PCR)

qRT-PCR was used to detect the expression of CTNNB1, the coding gene of β-catenin and the inflammatory factors TNF-α and IL-6. The extraction of total RNA of Caco-2 cells was performed using SteadyPure Universal RNA Extraction Kit II (Acres, Changsha, China) according to the manufacturer’s protocols. Genomic DNA was removed and then a reverse transcription kit (Acres, Changsha, China) was used to prepare cDNA. Finally, the commercial SYBR Green Pro Taq HS qPCR Kit (Acres, Changsha, China) was used for the polymerase chain reactions. The primers were synthesized by Sangon Biotech (Shanghai, China) and are shown in Table 1. The housekeeping gene β-actin was used as a reference for normalizing the relative mRNA expression. The relative expression levels of CTNNB1, TNF-α and IL-6 were calculated using the 2^−△△Ct^ method.

### 4.6. Western Blot

After exposure to PM2.5 for different times, cells were lysed in a RIPA buffer supplemented with protease and phosphatase inhibitors (Beyotime, Shanghai, China) following the provided protocol. Protein concentration was calculated using the BCA protein assay kit. The proteins were loaded onto and separated by a gel of 10% SDS/PAGE before transferring to a polyvinylidene fluoride (PVDF) membrane. The membrane was washed with Tris (hydroxymethyl) aminomethane-buffered saline (TBST), blocked with 5% milk at room temperature for 30 min, and then incubated with the appropriate primary antibody at 4 °C overnight. After incubation, the membrane was washed three times with TBST for 10 min each at room temperature and then incubated with HRP-conjugated secondary antibodies (anti-rabbit or anti-mouse IgG) for 1 h at room temperature. The protein bands were placed into a chemiluminescence instrument (Tianneng, Shanghai, China), and ECL reagent (Fude, Hangzhou, China) was added to visualize the bands. The grey value of the bands was quantified using Image J software (version 1.8.0).

### 4.7. Determination of Reactive Oxygen Species Levels

Intracellular ROS measurement was performed using an oxidation-sensitive fluorescent probe, namely, DCFH-DA following the manufacturer’s protocols. Caco-2 cells were seeded in 6-well plates at a density of 2.5 × 10^5^ cells per well and exposed to PM2.5 for 24 h. Subsequently, the cells were incubated with DCFH-DA (10 μM) at 37 °C for 20 min in the dark. After washing with DMEM three times, the cells were photographed under a fluorescence microscope (MaiAoDi, Fujian, China) and fluorescence intensity was analyzed using Image J software (version 1.8.0). Furthermore, the fluorescence intensity was also analyzed using a flow cytometer (BD, NJ, USA). Cells stained with DCFH-DA were trypsinized using EDTA-free trypsin (Gibco, NY, USA). The supernatant was discarded, and the cells were collected and resuspended in PBS for flow cytometer assay.

### 4.8. Malondialdehyde (MDA) Detection

MDA is a lipid oxidation end product, representing the oxidative stress in cells. It was measured using a commercial MDA assay kit (Beyotime, Shanghai, China). Cells were lysed on ice for 5 min. After centrifugation, the supernatant was collected as the sample for analysis. Protein concentrations of the samples were measured using a BCA protein assay kit. The absorbance was measured at 532 nm using a microplate reader, and MDA levels in the samples were calculated according to the manufacturer’s instructions.

### 4.9. Statistical Analysis

The experimental data were analyzed in GraphPad Prism 8.0.2. The results are presented as the mean ± SD from three experiments conducted independently. Data from different groups were compared using one-way ANOVA followed by the least significant difference (LSD) test. *p* < 0.05 was considered statistically significant.

## 5. Conclusions

These findings support the notion that inflammation, the PI3K/AKT signaling pathway, and oxidative stress may play critical roles in PM2.5-induced intestinal epithelial barrier damage. Our results partly expose the toxicological mechanisms by which PM2.5 caused intestinal damage. Based on these results, suppression of these targets with inhibitors could represent a promising therapeutic strategy in PM2.5-induced gastrointestinal diseases.

## Figures and Tables

**Table 1:** Primer sequence list.

Gene	Forward Primer (5′-3′)	Reverse Primer (5′-3′)
β-actin	GGACTTCGAGCAAGAGATGG	GGACTTCGAGCAAGAGATGG
CTNNB1	AAAGCGGCTGTTAGTCACTGG	CGAGTCATTGCATACTGTCCAT
IL-6	GCCTTCGGTCCAGTTGCCTTC	GTTCTGAAGAGGTGAGTGGCTGTC
TNF-α	CAATGGCGTGGAGCTGAGAGATAAC	TCTGGTAGGAGACGGCGATGC

## Data Availability

The original contributions presented in this study are included in the article; further inquiries can be directed to the corresponding author.
